# Long-term outcomes with frontline nilotinib versus imatinib in newly diagnosed chronic myeloid leukemia in chronic phase: ENESTnd 10-year analysis

**DOI:** 10.1038/s41375-020-01111-2

**Published:** 2021-01-07

**Authors:** Hagop M. Kantarjian, Timothy P. Hughes, Richard A. Larson, Dong-Wook Kim, Surapol Issaragrisil, Philipp le Coutre, Gabriel Etienne, Carla Boquimpani, Ricardo Pasquini, Richard E. Clark, Viviane Dubruille, Ian W. Flinn, Slawomira Kyrcz-Krzemien, Ewa Medras, Maria Zanichelli, Israel Bendit, Silvia Cacciatore, Ksenia Titorenko, Paola Aimone, Giuseppe Saglio, Andreas Hochhaus

**Affiliations:** 1 The University of Texas MD Anderson Cancer Center, Houston, TX USA; 2 South Australian Health and Medical Research Institute, Adelaide, SA Australia; 3 University of Adelaide, Adelaide, SA Australia; 4 Department of Medicine, Section of Hematology/Oncology, University of Chicago, Chicago, IL USA; 5 Seoul St. Mary’s Hospital, College of Medicine, Catholic University of Korea, Seoul, South Korea; 6 Faculty of Medicine, Siriraj Hospital, Mahidol University, Bangkok, Thailand; 7 Charité - Universitätsmedizin Berlin, Berlin, Germany; 8 Hematology Department, Institut Bergonié, Bordeaux, France; 9 Hemorio, Institute of Hematology, Rio de Janeiro, Brazil; 10 Oncoclínica Rio de Janeiro, Rio de Janeiro, Brazil; 11 Universidade Federal do Paraná, Hospital de Clinicas, Curitiba, Paraná Brazil; 12 Royal Liverpool University Hospital, Liverpool, United Kingdom; 13 Clinical Hematology, Nantes University Hospital, Nantes, France; 14 Sarah Cannon Research Institute, Nashville, TN USA; 15 Department of Hematology and Bone Marrow Transplantation, Medical University of Silesia, Katowice, Poland; 16 Department of Hematology, Blood Neoplasms and Bone Marrow Transplantation, Wroclaw Medical University, Wroclaw, Poland; 17 Instituto de Tratamento do Câncer Infantil, Instituto da Criança, Hospital das Clínicas, Universidade de São Paulo, São Paulo, Brazil; 18 Laboratory of Medical Investigation in Pathogenesis and Targeted Therapy in Onco-Immuno-Hematology (LIM-31), Department of Hematology, Hospital das Clínicas HCFMUSP, Faculdade de Medicina, Universidade de São Paulo, São Paulo, Brazil; 19 Novartis Pharma AG, Basel, Switzerland; 20 Novartis Pharmaceuticals Corporation, Moscow, Russian Federation; 21 Division of Internal Medicine and Hematology, University of Turin, Turin, Italy; 22 Universitätsklinikum Jena, Jena, Germany

**Keywords:** Chronic myeloid leukaemia, Haematological cancer

## Abstract

In the ENESTnd study, with ≥10 years follow-up in patients with newly diagnosed chronic myeloid leukemia (CML) in chronic phase, nilotinib demonstrated higher cumulative molecular response rates, lower rates of disease progression and CML-related death, and increased eligibility for treatment-free remission (TFR). Cumulative 10-year rates of MMR and MR^4.5^ were higher with nilotinib (300 mg twice daily [BID], 77.7% and 61.0%, respectively; 400 mg BID, 79.7% and 61.2%, respectively) than with imatinib (400 mg once daily [QD], 62.5% and 39.2%, respectively). Cumulative rates of TFR eligibility at 10 years were higher with nilotinib (300 mg BID, 48.6%; 400 mg BID, 47.3%) vs imatinib (29.7%). Estimated 10-year overall survival rates in nilotinib and imatinib arms were 87.6%, 90.3%, and 88.3%, respectively. Overall frequency of adverse events was similar with nilotinib and imatinib. By 10 years, higher cumulative rates of cardiovascular events were reported with nilotinib (300 mg BID, 16.5%; 400 mg BID, 23.5%) vs imatinib (3.6%), including in Framingham low-risk patients. Overall efficacy and safety results support the use of nilotinib 300 mg BID as frontline therapy for optimal long-term outcomes, especially in patients aiming for TFR. The benefit-risk profile in context of individual treatment goals should be carefully assessed.

## Introduction

Nilotinib is a second-generation BCR-ABL1 tyrosine kinase inhibitor (TKI) widely used for the treatment of patients with newly diagnosed Philadelphia chromosome–positive (Ph+) chronic myeloid leukemia in chronic phase (CML-CP) or imatinib-resistant or imatinib-intolerant Ph+ CML in CP or accelerated phase (AP) [1–3]. The approved nilotinib dose for adult patients with newly diagnosed Ph+ CML-CP is 300 mg twice-daily, and for those with resistant or intolerant Ph+ CML-CP and CML-AP, the dose is 400 mg twice-daily [2, 3].

Throughout the first 5 years of follow-up in the pivotal phase 3 ENESTnd (Evaluating Nilotinib Efficacy and Safety in Clinical Trials–Newly Diagnosed Patients) study, treatment with nilotinib resulted in higher rates of major molecular response (MMR; *BCR-ABL1* ≤ 0.1% on the International Scale [*BCR-ABL1*^IS^]) and deep molecular response (DMR; including MR^4^ [*BCR-ABL1*^IS^ ≤ 0.01%] and MR^4.5^ [*BCR-ABL1*^IS^ ≤ 0.0032%]) over imatinib as frontline therapy for newly diagnosed CML-CP [4, 5]. In the primary endpoint analysis, rates of MMR at 12 months were 44% with nilotinib 300-mg twice-daily, 43% with nilotinib 400-mg twice-daily, and 22% with imatinib (400-mg once-daily, *P* < 0.001 for both comparisons) [4]. With 5 years of follow-up, 54% of patients on nilotinib 300-mg twice-daily and 52% of patients on nilotinib 400-mg twice-daily achieved MR^4.5^ compared with 31% of patients on imatinib (*P* < 0.0001 for both comparisons) [5]. Lower rates of progression to AP/blast phase (BP) with nilotinib versus imatinib were observed throughout the first 5 years of follow-up [5].

The 5-year ENESTnd analysis showed a higher frequency of cardiovascular events (CVEs) with nilotinib than with imatinib, particularly in the nilotinib 400-mg twice-daily arm. Baseline Framingham general cardiovascular risk scores were predictive of patients’ risk of developing a CVE during nilotinib therapy, suggesting that patients at risk of developing CVEs during therapy with TKIs might be identifiable at baseline and that active monitoring and treatment of comorbidities and cardiovascular risk factors in all patients are needed [5]. Overall, the benefit-risk profile of nilotinib as frontline therapy for patients with CML-CP remained positive [4–8].

Patients with CML on TKI therapy have a life expectancy comparable to that of the general population [9, 10] and are likely to continue treatment for many years, possibly decades [11, 12]. Patients may develop comorbidities with age [5, 13–15] and may also develop distinct, long-term, TKI therapy–related adverse events (AEs) [5, 16]. Managing comorbidities and AEs is an important aspect of long-term treatment [5, 10, 11]. For patients achieving sustained DMR, treatment-free remission (TFR) may be an additional treatment goal [10, 12, 17].

To allow a comprehensive assessment of the long-term benefits and risks of nilotinib and imatinib in patients with CML-CP, the final results from ENESTnd after ≥10 years of follow-up are reported here.

## Methods

### Study design and patients

Study design and eligibility criteria for ENESTnd (NCT00471497) have been described previously [4–8]. Briefly, patients with newly diagnosed CML-CP were randomized to receive nilotinib 300-mg twice-daily (*n* = 282), nilotinib 400-mg twice-daily (*n* = 281), or imatinib 400-mg once-daily (*n* = 283) in the core phase. Some patients who discontinued their assigned core treatment due to suboptimal response, treatment failure, or progressive disease could enter the extension phase of the study (Supplementary Fig. 1; Supplementary Table 1) [6].

### Study endpoints and assessments

Long-term endpoints included cumulative rates of MMR, MR^4^, and MR^4.5^, progression to AP/BP, overall survival (OS), progression-free survival (PFS), and safety. Molecular responses were assessed by *BCR-ABL1/ABL1* transcript ratios using real-time quantitative polymerase chain reaction (RT-qPCR) at a central laboratory (MolecularMD, Portland, OR, USA) standardized to the IS. Data on progression to AP/BP and survival were prospectively collected as described previously [5]. Time to progression to AP/BP was defined as time from randomization until progression to AP/BP or death due to advanced CML, whichever occurred first. Death due to advanced CML was defined as any death (at any time) for which the principal cause was reported by the investigator as “study indication” (i.e., due to CML) or, if subsequent to documented progression to AP/BP, any death for which the cause was reported as “unknown” or was not reported. OS was defined as time from randomization until death due to any cause. PFS was defined as time from randomization until progression to AP/BP or death due to any cause. Rates of freedom from progression to AP/BP, PFS, and OS on study considered events that occurred during core or extension treatment and those that occurred during follow-up after discontinuation of core or extension treatment. Further details on the study protocol and assessment schedule have been published previously [4, 5].

### Statistical analysis

The efficacy and safety data presented here are based on a final analysis of the study following the last patient’s last visit (21 August 2019), when all patients had completed ≥10 years of treatment (in the core or extension phase) or discontinued earlier. All analyses presented here included data from the core phase only, with the exception of survival and progression “on study” analyses, which also included data from the extension phase. Data from the extension phase are reported based on treatment initially assigned to patients during core phase. For analyses combining data from the 2 nilotinib arms, extension phase data for patients who switched from nilotinib 300-mg twice-daily to nilotinib 400-mg twice-daily were also included.

**Fig. 1:**
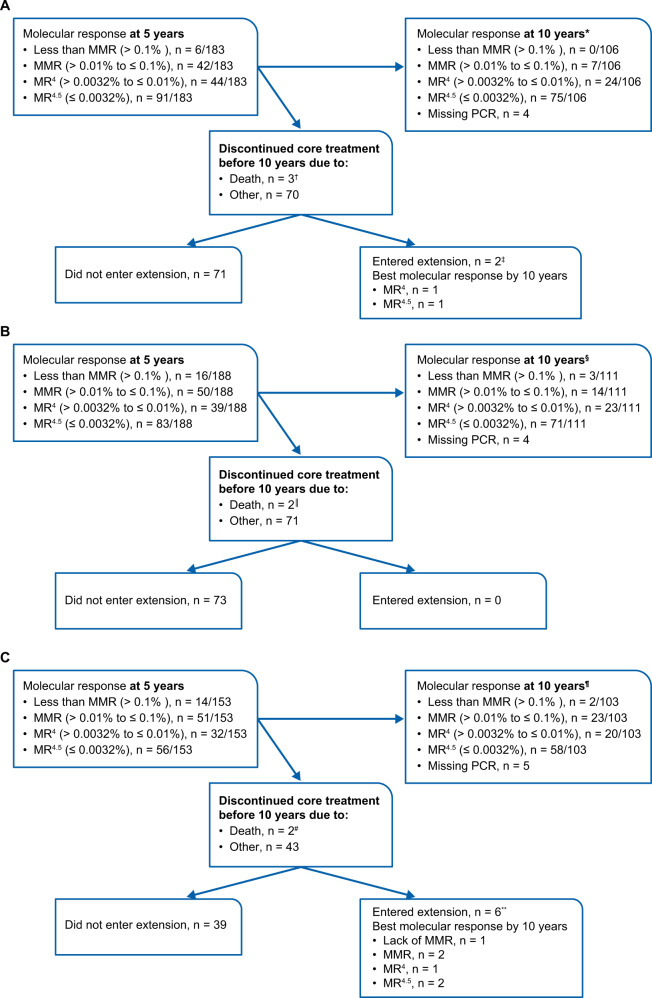
Change in molecular response from 5 years to 10 years by molecular response at 5 years in patients with evaluable RQ-PCR at 5 years. **A** Nilotinib 300 mg twice daily. **B** Nilotinib 400 mg twice daily. **C** Imatinib 400 mg twice daily. ^*^Of patients without MMR at 5 years, 33.3% (2/6) achieved MMR or better at 10 years. Of patients without MR^4^ at 5 years, 43.8% (21/48) achieved MR^4^ or better at 10 years. Of patients without MR^4.5^ at 5 years, 38.0% (35/92) achieved MR^4.5^ at 10 years. ^†^All 3 patients were in MR^4.5^ at 5 years but died before 10 years (1 due to cardiac arrest, 1 due to multiple organ dysfunction syndrome, and 1 due to pneumonia) while still in MR^4.5^. ^‡^Both patients were in MR^4.5^ at 5 years but were not in MMR before entering the extension phase. ^§^Of patients without MMR at 5 years, 43.8% (7/16) achieved MMR or better at 10 years. Of patients without MR^4^ at 5 years, 37.9% (25/66) achieved MR^4^ or better at 10 years. Of patients without MR^4.5^ at 5 years, 28.6% (30/105) achieved MR^4.5^ at 10 years. ^||^One patient was in MR^4.5^ at 5 years and in MR^4^ before death due to myocardial infarction. The other patient was in MR^4^ at 5 years and before death due to an unknown reason. ^¶^Of patients without MMR at 5 years, 50% (7/14) achieved MMR or better at 10 years. Of patients without MR^4^ at 5 years, 33.8% (22/65) achieved MR^4^ or better at 10 years. Of patients without MR^4.5^ at 5 years, 22.7% (22/97) achieved MR^4.5^ at 10 years. ^#^Both patients were at MMR at 5 years but died before 10 years (1 due to cardiac arrest and 1 due to pneumonia), while still in MMR. ^**^Two patients achieved MR^4.5^, 1 achieved MR^4^, 2 achieved MMR, and 1 had responses less than MMR in the extension phase.

Efficacy analyses included the intent-to-treat population (all randomized patients; *N* = 846). Analysis of molecular response at 10 years according to molecular response at 5 years included only patients with typical *BCR-ABL1* transcripts at baseline who remained on treatment at 5 years based on a 3-month time window (nilotinib 300-mg twice-daily arm, *n* = 183; nilotinib 400-mg twice-daily arm, *n* = 188; imatinib arm, *n* = 153) (Fig. 1). Response rates were compared using the Cochran–Mantel–Haenszel test stratified by Sokal risk group. Clopper–Pearson 95% 2-sided CIs for response rates are shown. Time-to-event variables were analyzed using the Kaplan–Meier (KM) method and were compared between groups using log-rank tests stratified by Sokal risk group. Hazard ratios and 95% 2-sided CIs were derived from a Cox model stratified by Sokal risk group; 95% CIs for KM estimates were derived using the standard error calculated with Greenwood’s formula. Nominal 2-sided *P* values, when provided, are for descriptive purposes only without multiplicity adjustments; therefore, no formal statistical claim can be made, and statistical interpretation should be made with caution. Safety analyses included all patients who received ≥1 dose of study drug (nilotinib 300-mg twice-daily arm, *n* = 279; nilotinib 400-mg twice-daily arm, *n* = 277; imatinib arm, *n* = 280). CVE rates for each treatment group were determined by using cumulative events over time as well as the KM method. Analyses were also performed by combining data from both nilotinib arms to compare responses and outcomes achieved with nilotinib (*n* = 563) versus imatinib (*n* = 283), regardless of dose. The extent of exposure was analyzed comparing exposures in the combined safety population of both nilotinib arms (*n* = 556) versus the imatinib arm (*n* = 280).

Eligibility for TFR, per criteria used in ENESTfreedom [18], was defined as achieving MR^4.5^ or better after ≥2 years of treatment and then maintaining sustained DMR for ≥1 year (defined as having no RT-qPCR assessment showing a response level worse than MR^4^, a maximum of 2 assessments with response level of MR^4^, and MR^4.5^ or better in the last assessment).

## Results

### Patients

By the date of the last patient’s last visit, 107 (37.9%), 99 (35.2%), and 99 (35.0%) patients in the nilotinib 300-mg twice-daily, nilotinib 400-mg twice-daily, and imatinib arms, respectively, completed the study and were treated for ≥10 years overall. The most common reasons for discontinuation from core treatment in all 3 arms included AEs, withdrawal of consent, and suboptimal response or treatment failure (Supplementary Table 1). The median duration of core treatment was 82.8, 87.5, and 64.0 months, respectively, in the nilotinib 300-mg twice-daily, nilotinib 400-mg twice-daily, and imatinib arms. Twenty-six (9.2%), 3 (1.1%), and 48 (17.0%) patients, respectively, discontinued core treatment and entered the extension phase of the study. Among the 77 patients who entered the extension phase, 12, 2, and 21, respectively, completed their planned treatment, and 14, 1, and 27 discontinued early.

One hundred seventy-one (61.3%), 182 (65.7%), and 146 (52.1%) patients, respectively, in the nilotinib 300-mg twice-daily, nilotinib 400-mg twice-daily, and imatinib arms remained on treatment for ≥5 years; and 101 (36.2%), 87 (31.4%), and 90 (32.1%) patients, respectively, received treatment for ≥10 years.

### Long-term outcomes

Fewer progressions to AP/BP were observed with nilotinib than with imatinib (Table 1). A total of 6, 4, and 11 progressions to AP and 6, 6, and 14 progressions to BP were reported on study in the nilotinib 300-mg twice-daily, nilotinib 400-mg twice-daily, and imatinib arms, respectively. Among those who progressed to BP, 2, 3, and 4 patients, respectively, had progressed to AP first. Overall, a total of 11, 7, and 24 progressions to AP/BP, including CML-related deaths, were reported on study in the nilotinib 300-mg twice-daily, nilotinib 400-mg twice-daily, and imatinib arms, respectively, including 1, 1, and 5, respectively, after the first 5 years, all of which occurred during the follow-up period after core or extension treatment discontinuation due to reasons other than progression. After the 5-year analysis, no new progressions to AP/BP on core treatment were observed.

**Table 1:** Progression to AP/BP.

	Nilotinib 300 mg twice daily	Nilotinib 400 mg twice daily	Imatinib 400 mg once daily
Progression to AP/BP on study, *n*^**a**^	282	281	283
Progression to AP/BP, *n*^b^	11	7	24
Estimated rate of freedom from progression to AP/BP, % (95% CI)			
At 5 years	96.3 (94.1–98.6)	97.8 (96.0–99.5)	92.2 (89.1–95.4)
At 10 years	95.9 (93.5–98.3)	97.3 (95.3–99.3)	90.8 (87.3–94.3)
HR vs imatinib (95% CI)	0.45 (0.22–0.92)	0.28 (0.12–0.66)	NA
* P* vs imatinib	0.02	<0.005	NA
Progression to AP, *n*^c^	6	4	11
Estimated rate of freedom from progression to AP, % (95% CI)			
At 5 years	98.2 (96.6–99.8)	98.5 (97.0–100)	97.1 (95.1–99.1)
At 10 years	97.7 (95.9–99.5)	98.5 (97.0–100)	95.5 (92.9–98.2)
HR vs imatinib (95% CI)	0.54 (0.20–1.46)	0.36 (0.11–1.12)	NA
* P* vs imatinib	0.22	0.07	NA
Progression to BP, *n*^c^	6	6	14
Estimated rate of freedom from progression to BP, % (95% CI)			
At 5 years	97.8 (96.0–99.5)	98.1 (96.5–99.8)	94.9 (92.3–97.5)
At 10 years	97.8 (96.0–99.5)	97.7 (95.8–99.5)	94.9 (92.3–97.5)
HR vs imatinib (95% CI)	0.42 (0.16–1.09)	0.41 (0.16–1.08)	NA
* P* vs imatinib	0.06	0.06	NA
Progression to AP/BP on study by age at baseline			
Patients <60 years, *n*^a^	223	228	224
Progression to AP/BP in patients <60 years, *n*	7	6	20
Estimated rate of freedom from progression to AP/BP at 10 years, % (95% CI)	96.7 (94.3–99.1)	97.2 (94.9–99.4)	90.4 (86.3–94.4)
Patients ≥60 years, *n*^a^	59	53	59
Progression to AP/BP in patients ≥60 years, *n*	4	1	4
Estimated rate of freedom from progression to AP/BP at 10 years, % (95% CI)	92.4 (85.2–99.6)	98.0 (94.1–100)	92.5 (85.3–99.6)
Progression to AP/BP on study by Sokal risk score at baseline			
Patients in low Sokal risk group, *n*^a^	103	103	104
Low Sokal risk, *n*	2	1	1
Estimated rate of freedom from progression to AP/BP at 10 years, % (95% CI)	97.8 (94.8–100)	99.0 (97.1–100)	98.8 (96.4–100)
Patients in intermediate Sokal risk group, *n*^a^	101	100	101
Intermediate Sokal risk, *n*	2	1	10
Estimated rate of freedom from progression to AP/BP at 10 years, % (95% CI)	98.0 (95.3–100)	99.0 (96.9–100)	89.9 (84.0–95.8)
Patients in high Sokal risk group, *n*^a^	78	78	78
High Sokal risk, *n*	7	5	13
Estimated rate of freedom from progression to AP/BP at 10 years, % (95% CI)	90.3 (83.5–97.2)	92.6 (86.3–98.9)	80.9 (71.4–90.4)

NA indicates not applicable.

^a^The evaluable n for each analysis.

^b^Includes progressions and CML-related deaths.

^c^Includes progressions only.

The estimated 10-year rates of freedom from progression to AP/BP were higher in the nilotinib arms than the imatinib arm among patients <60 years old at baseline and were comparable across arms in patients ≥60 years old at baseline. In an analysis according to baseline Sokal risk score, the estimated 10-year rates of freedom from progression to AP/BP were comparable across arms in the low Sokal risk group and higher in the nilotinib arms in the intermediate and high Sokal risk groups (Table 1).

At 10 years, the rates of PFS and OS on study were similar across the 3 arms (Supplementary Fig. 2) and were also comparable in the analysis conducted by Sokal score, with the highest rates reported in patients with low Sokal risk (Tables 2 and 3).

**Table 2:** Estimated 10-year OS rates.

	Nilotinib 300 mg twice daily	Nilotinib 400 mg twice daily	Imatinib 400 mg once daily
OS			
All patients, *n*	282	281	283
OS events, *n*	32	24	29
Estimated rate of OS, % (95% CI)			
At 5 years	93.7 (90.8–96.6)	96.3 (94.0–98.5)	91.8 (88.5–95.1)
At 10 years	87.6 (83.5–91.7)	90.3 (86.5–94.1)	88.3 (84.2–92.4)
HR vs imatinib (95% CI)	1.07 (0.64–1.76)	0.79 (0.46–1.36)	NA
* P* vs imatinib	0.80	0.40	NA
OS on study by age at baseline			
Patients <60 years, *n*^a^	223	228	224
OS events in patients <60 years, *n*	15	11	21
Estimated rate of OS at 10 years, % (95% CI)	92.7 (89.1–96.3)	94.5 (91.3–97.7)	89.7 (85.5–93.9)
Patients ≥60 years, *n*^a^	59	53	59
OS events in patients ≥60 years, *n*	17	13	8
Estimated rate of OS at 10 years, % (95% CI)	67.0 (53.7–80.4)	69.4 (54.6–84.2)	83.4 (72.8–94.1)
OS on study by Sokal risk score at baseline			
Patients in low Sokal risk group, *n*^a^	103	103	104
OS events, *n*	5	5	1
Estimated rate of OS at 10 years, % (95% CI)	94.7 (90.3–99.2)	94.1 (89.1–99.1)	98.8 (96.4–100)
Patients in intermediate Sokal risk group, *n*^a^	101	100	101
OS events, *n*	12	9	14
Estimated rate of OS at 10 years, % (95% CI)	88.0 (81.3–94.7)	89.6 (83.2–96.1)	84.3 (76.6–92.1)
Patients in high Sokal risk group, *n*^a^	78	78	78
OS events, *n*	15	10	14
Estimated rate of OS at 10 years, % (95% CI)	76.5 (65.9–87.1)	85.9 (77.3–94.6)	78.9 (68.8–88.9)

NA indicates not applicable.

^a^The evaluable *n* for each analysis.

In an analysis of 10-year outcomes according to age at baseline, the estimated 10-year OS rates in the nilotinib 300-mg twice-daily, nilotinib 400-mg twice-daily, and imatinib arms were 92.7%, 94.5%, and 89.7%, respectively, among patients <60 years old and 67.0%, 69.4%, and 83.4%, respectively, among patients ≥60 years old (Table 2). The estimated 10-year PFS rates on study were 91.8%, 94.1%, and 88.3%, respectively, among patients <60 years old and 63.7%, 69.4%, and 83.6%, respectively, among patients ≥60 years old (Table 3).

**Table 3:** Estimated 10-year PFS rates.

	Nilotinib 300 mg twice daily	Nilotinib 400 mg twice daily	Imatinib 400 mg once daily
PFS on study			
All patients, *n*	282	281	283
PFS events, *n*	36	25	32
Estimated rate of PFS, % (95% CI)			
At 5 years	92.3 (89.1–95.4)	95.9 (93.5–98.3)	91.2 (87.8–94.5)
At 10 years	86.2 (81.9–90.5)	89.9 (86.1–93.8)	87.2 (83.0–91.4)
HR vs imatinib (95% CI)	1.08 (0.67–1.74)	0.74 (0.44–1.25)	NA
* P* vs imatinib	0.75	0.27	NA
PFS on study by age at baseline			
Patients <60 years, *n*^a^	223	228	224
PFS events in patients <60 years, *n*	17	12	24
Estimated rate of PFS at 10 years, % (95% CI)	91.8 (88.0–95.5)	94.1 (90.8–97.3)	88.3 (83.9–92.8)
Patients ≥60 years, *n*^a^	59	53	59
PFS events in patients ≥60 years, *n*	19	13	8
Estimated rate of PFS at 10 years, % (95% CI)	63.7 (50.2–77.3)	69.4 (54.5–84.2)	83.6 (73.0–94.2)
PFS on study by Sokal risk score at baseline			
Patients in low Sokal risk group, *n*^a^	103	103	104
PFS events, *n*	6	5	2
Estimated rate of PFS at 10 years, % (95% CI)	93.7 (88.9–98.6)	94.1 (89.1–99.1)	97.5 (94.2–100)
Patients in intermediate Sokal risk group, *n*^a^	101	100	101
PFS events, *n*	13	9	15
Estimated rate of PFS at 10 years, % (95% CI)	87.1 (80.2–93.9)	89.6 (83.2–96.1)	83.6 (75.8–91.4)
Patients in high Sokal risk group, *n*^a^	78	78	78
PFS events, *n*	17	11	15
Estimated rate of PFS at 10 years, % (95% CI)	74.0 (63.2–84.8)	84.6 (75.7–93.6)	77.7 (67.6–87.9)

NA indicates not applicable.

^a^The evaluable *n* for each analysis.

A total of 32, 23, and 29 deaths on study from any cause were reported in the nilotinib 300-mg twice-daily, nilotinib 400-mg twice-daily, and imatinib arms, respectively (Supplementary Table 2). One patient randomized to the imatinib arm died due to CML before the first treatment dose. Of all deaths on study, 16, 14, and 11 in the nilotinib 300-mg twice-daily, nilotinib 400-mg twice-daily, and imatinib arms, respectively, occurred after 5 years. The most common causes of death overall were CML, general disorders, and infections. The total number of deaths due to CML was 6, 5, and 15 in the nilotinib 300-mg twice-daily, nilotinib 400-mg twice-daily, and imatinib arms, respectively. Six deaths occurred on study due to CVEs, 3 in the nilotinib 300-mg twice-daily arm and 3 in the nilotinib 400-mg twice-daily arm, of which 2 and 3, respectively, occurred after 5 years. Two of the deaths due to CVEs occurred during or within 28 days of discontinuing core study treatment; 1 was due to cerebrovascular accident in the nilotinib 300-mg twice-daily arm, and 1 was due to myocardial infarction in the nilotinib 400-mg twice-daily arm.

### Molecular response

**Fig. 2:**
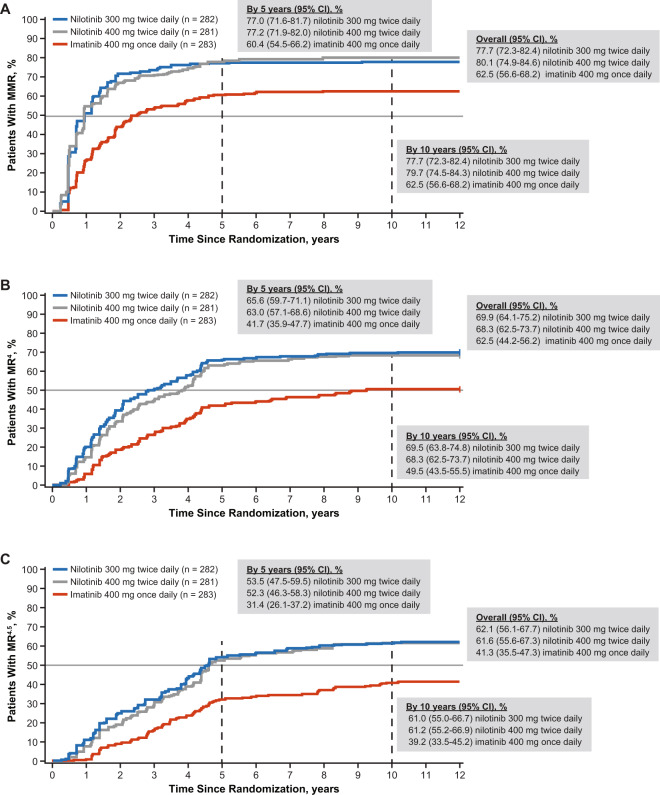
Cumulative molecular response rates. Cumulative proportion of patients with (**A**) major molecular response (MMR; *BCR-ABL1*^IS^ ≤ 0.1%), (**B**) MR^4^ (*BCR-ABL1*^IS^ ≤ 0.01%), and (**C**) MR^4.5^ (*BCR-ABL1*^IS^ ≤ 0.0032%). Cumulative MMR, MR^4^, and MR^4.5^ results are an analysis of data from the core phase.

By 10 years, cumulative MMR, MR^4^, and MR^4.5^ rates were higher with nilotinib than with imatinib (Fig. 2).

Change in molecular response from 5 to 10 years by molecular response at 5 years among the patients still on treatment at 5 years is shown in Fig. 1. In the nilotinib 300-mg twice-daily arm, 6, 48, and 92 evaluable patients were not in MMR, MR^4^, and MR^4.5^, respectively, at 5 years while on treatment. Of those patients, 33.3% (2/6), 43.8% (21/48), and 38.0% (35/92) achieved MMR, MR^4^, and MR^4.5^, respectively, at 10 years while continuing the same treatment. In the nilotinib 400-mg twice-daily arm, 16, 66, and 105 evaluable patients were not in MMR, MR^4^, and MR^4.5^, respectively, at 5 years while on treatment. Of those patients, 43.8% (7/16), 37.9% (25/66), and 28.6% (30/105) achieved MMR, MR^4^, and MR^4.5^, respectively, at 10 years while continuing the same treatment. In the imatinib arm, 14, 65, and 97 evaluable patients were not in MMR, MR^4^, and MR^4.5^, respectively, at 5 years while on treatment. Of those patients, 50% (7/14), 33.8% (22/65), and 22.7% (22/97) achieved MMR, MR^4^, and MR^4.5^, respectively, at 10 years while continuing the same treatment.

### Combined analysis

After combining data from the 2 nilotinib arms, 368 (66.2%) and 146 (52.1%) patients, respectively, in either nilotinib arm and in the imatinib arm remained on treatment for ≥5 years, and 199 (35.8%) and 90 (32.1%) patients, respectively, remained on the same treatment for ≥10 years, regardless of dose. Rates of MMR and MR^4.5^ were higher with nilotinib at 5 years (MMR, 63.6%; MR^4.5^, 31.1%) and 10 years (MMR, 40.7%; MR^4.5^, 27.0%) than with imatinib at 5 years (MMR, 49.1%; MR^4.5^, 19.8%) and 10 years (MMR, 36.4%; MR^4.5^, 21.2%). The median time to first MMR was shorter with nilotinib (8.41 months; range, 1.9–115.9) than with imatinib (14.16 months; range, 2.8–95.6). Similarly, the median time to first MR^4.5^ was shorter with nilotinib (37.65 months; range, 2.8–122.1) than with imatinib (41.63 months; range, 7.5–122.6).

### TFR eligibility

By 10 years, more patients in each nilotinib arm than in the imatinib arm achieved sustained DMR (Supplementary Table 3). Rates of sustained DMR were analyzed in patient subsets based on Sokal risk at baseline, 3-month molecular response levels, and time to first MR^4.5^. In each Sokal risk group, rates of sustained DMR were higher with nilotinib than with imatinib.

In all 3 study arms, a higher proportion of patients with *BCR-ABL1*^IS^ ≤ 10% at 3 months achieved sustained DMR compared with those who had *BCR-ABL1*^IS^ > 10% at 3 months; moreover, more patients in the nilotinib arms versus the imatinib arm achieved *BCR-ABL1*^IS^ ≤ 10% at 3 months, and the rate of sustained DMR among patients who achieved *BCR-ABL1*^IS^ ≤ 10% at 3 months was higher in the nilotinib arms versus the imatinib arm. In all arms, rates of sustained DMR were high among patients who achieved MR^4.5^ at any time; however, more patients in the nilotinib arms than the imatinib arm achieved MR^4.5^, and they did so earlier.

**Fig. 3:**
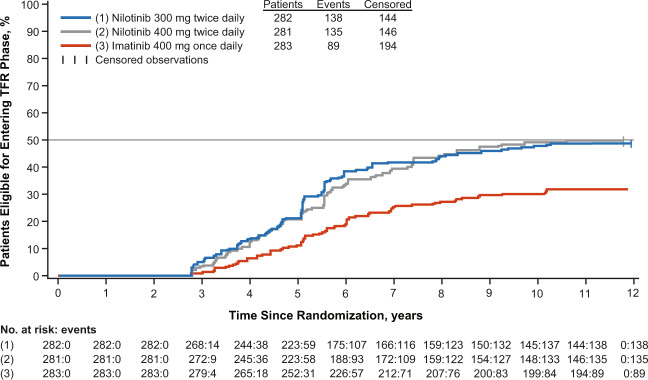
Cumulative rate of TFR eligibility. Patients were considered eligible for TFR if they achieved MR^4.5^ or better in any RQ-PCR assessment after ≥2 years and then maintained sustained DMR for ≥1 year, during which time no RQ-PCR assessment showed a response level worse than MR^4^, a maximum of 2 assessments occurred between MR^4^ and MR^4.5^, and the last assessment showed MR^4.5^ or better.

The estimated cumulative rates of TFR eligibility (estimated using ENESTfreedom criteria [18]) with nilotinib 300-mg twice-daily, nilotinib 400-mg twice-daily, and imatinib, respectively, at 5 years were 20.9% (95% CI, 16.2–25.7%), 20.6% (15.9–25.4%), and 11.0% (7.3–14.6%) and 10 years were 48.6% (42.7–54.4%), 47.3% (41.5–53.2%), and 29.7% (24.4–35.0%) (Fig. 3).

### Safety

The most common nonhematologic AEs of any cause in both nilotinib arms were rash and headache, while diarrhea and nausea were most common in the imatinib arm. In all 3 arms, most nonhematologic AEs were grade 1/2. Newly occurring or worsening grade 3/4 hematologic abnormalities were more common (specifically, decreased absolute lymphocytes and neutrophils) with imatinib than with nilotinib. Among the most common newly occurring or worsening grade 3/4 biochemical abnormalities, elevations in glucose occurred in 24 (8.6%), 21 (7.6%), and 1 (0.4%) patients in the nilotinib 300-mg twice-daily, nilotinib 400-mg twice-daily, and imatinib arms, respectively; newly occurring or worsening grade 3/4 lipase elevations occurred in 29 (10.4%), 30 (10.8%), and 12 (4.3%) patients, respectively (Supplementary Table 4).

Adverse events of interest such as severe fluid retention (including pleural effusion, pericardial effusion, and pulmonary edema) and pancreatitis were infrequent (<4% of patients in all arms). Any-grade hepatotoxicity occurred more frequently in the nilotinib arms (300-mg twice-daily, 48.4%; 400-mg twice-daily, 53.1%) than in the imatinib arm (17.5%), and any-grade electrocardiogram QT prolongation was also more common with nilotinib (300-mg twice-daily, 6.8%; 400-mg twice-daily, 7.9%) than with imatinib (3.9%); rates of any-grade and grade 3/4 AE groups of interest are shown in Supplementary Table 5.

CVEs were reported in 46 (16.5%), 65 (23.5%), and 10 (3.6%) patients, respectively, in the nilotinib 300-mg twice-daily, nilotinib 400-mg twice-daily, and imatinib arms (Table 4). KM-estimated CVE rates were 10.6%, 17.9%, and 3.2%, respectively, at 5 years and continued to increase to 24.8%, 33.4%, and 6.3%, respectively, at 10 years; however, the incidence of newly occurring CVEs displayed in a yearly time window seemed similar across years in all arms among patients on long-term treatment (Supplementary Fig. 3).

**Table 4:** Cardiovascular events (CVEs).

	Nilotinib 300 mg twice daily	Nilotinib 400 mg twice daily	Imatinib 400 mg once daily
CVEs, *n*^a^	279	277	280
Cumulative CVEs			
All CVEs	46 (16.5)	65 (23.5)	10 (3.6)
Ischemic heart disease	22 (7.9)	36 (13.0)	8 (2.9)
Peripheral arterial occlusive disease	18 (6.5)	20 (7.2)	0
Ischemic cerebrovascular disease	13 (4.7)	21 (7.6)	1 (0.4)
Other CVEs	4 (1.4)	4 (1.4)	1 (0.4)
CVEs by age at baseline			
Patients < 60 years, *n*^b^	221	227	221
All CVEs	26 (11.8)	48 (21.1)	5 (2.3)
Ischemic heart disease	12 (5.4)	27 (11.9)	5 (2.3)
Peripheral arterial occlusive disease	9 (4.1)	15 (6.6)	0
Ischemic cerebrovascular disease	7 (3.2)	16 (7.0)	0
Other CVEs	3 (1.4)	3 (1.3)	0
Patients ≥ 60 years, *n*^b^	58	50	59
All CVEs	20 (34.5)	17 (34.0)	5 (8.5)
Ischemic heart disease	10 (17.2)	9 (18.0)	3 (5.1)
Peripheral arterial occlusive disease	9 (15.5)	5 (10.0)	0
Ischemic cerebrovascular disease	6 (10.3)	5 (10.0)	1 (1.7)
Other CVEs	1 (1.7)	1 (2.0)	1 (1.7)

Values are *n* (%) unless otherwise noted.

^a^A patient with multiple occurrences of a CVE is counted only once in the CVE subcategory.

^b^The evaluable *n* for each analysis.

To determine the impact of preexisting cardiovascular risk on the development of CVEs during nilotinib or imatinib treatment, baseline Framingham general cardiovascular risk scores [19] were calculated for patients with no history of CVEs prior to study entry (nilotinib 300-mg twice-daily, *n* = 259; nilotinib 400-mg twice-daily, *n* = 266; imatinib, *n* = 264). The majority of patients were in the low-risk category. Similar to trends observed before 5 years [5], in patients on treatment for >5 years, baseline Framingham general cardiovascular risk scores were predictive of patients’ risk of developing a CVE during treatment. In patients on treatment for >5 years, CVEs occurred more frequently among patients in the high-risk (nilotinib 300-mg twice-daily, 33.3%; nilotinib 400-mg twice-daily, 33.3%; imatinib, 4.8%) and intermediate-risk (28.0%, 48.5%, 17.4%, respectively) categories in each arm than in the low-risk category (8.7%, 15.2%, 1.1%, respectively). In the low-risk category, CVE rates after 5 years were higher than the rates before 5 years (2.2%, 4.0%, 0.5%, respectively) (Table 5).

**Table 5:** Cardiovascular events (CVEs) before 5 years and after 5 years by Framingham risk category at baseline.

	Framingham general risk of CVE < 10%			Framingham general risk of CVE ≥ 10% to <20%			Framingham general risk of CVE ≥ 20%		
	Nilotinib 300 mg twice daily	Nilotinib 400 mg twice daily	Imatinib 400 mg once daily	Nilotinib 300 mg twice daily	Nilotinib 400 mg twice daily	Imatinib 400 mg once daily	Nilotinib 300 mg twice daily	Nilotinib 400 mg twice daily	Imatinib 400 mg once daily
CVEs occurring before 5 years									
All patients, *n*	178	176	182	41	52	49	40	38	33
All CVEs	4 (2.2)	7 (4.0)	1 (0.5)	5 (12.2)	10 (19.2)	2 (4.1)	6 (15.0)	11 (28.9)	1 (3.0)
Ischemic heart disease	3 (1.7)	5 (2.8)	1 (0.5)	3 (7.3)	6 (11.5)	1 (2.0)	2 (5.0)	5 (13.2)	1 (3.0)
Peripheral arterial occlusive disease	1 (0.6)	0	0	1 (2.4)	1 (1.9)	0	3 (7.5)	5 (13.2)	0
Ischemic cerebrovascular disease	0	1 (0.6)	0	1 (2.4)	4 (7.7)	1 (2.0)	1 (2.5)	1 (2.6)	0
Other CVEs	0	1 (0.6)	0	1 (2.4)	0	0	2 (5.0)	1 (2.6)	0
CVEs occurring after 5 years									
Patients on treatment for > 5 years, *n*	115	125	94	25	33	23	24	21	21
All CVEs	10 (8.7)	19 (15.2)	1 (1.1)	7 (28.0)	16 (48.5)	4 (17.4)	8 (33.3)	7 (33.3)	1 (4.8)
Ischemic heart disease	4 (3.5)	11 (8.8)	1 (1.1)	3 (12.0)	7 (21.2)	2 (8.7)	2 (8.3)	2 (9.5)	1 (4.8)
Peripheral arterial occlusive disease	2 (1.7)	6 (4.8)	0	3 (12.0)	3 (9.1)	0	4 (16.7)	6 (28.6)	0
Ischemic cerebrovascular disease	4 (3.5)	6 (4.8)	0	1 (4.0)	7 (21.2)	1 (4.3)	2 (8.3)	3 (14.3)	0
Other CVEs	0	0	0	0	1 (3.0)	1 (4.3)	0	1 (4.8)	0

Values are *n* (%) unless otherwise noted.

## Discussion

With 10 years of follow-up in ENESTnd, the balance of benefits and risks of nilotinib versus imatinib in patients with newly diagnosed CML-CP can be thoroughly evaluated. Nilotinib demonstrated benefits over imatinib in several clinical patient outcomes, including higher cumulative molecular response rates, lower rates of progression to AP/BP and CML-related deaths, and increased eligibility for TFR.

The cumulative rates of MMR, MR^4^, and MR^4.5^ were higher by 5 years and by 10 years with nilotinib than with imatinib. In the combined analysis of the 2 nilotinib arms, response rates were higher at 5 years and 10 years with nilotinib versus imatinib, and patients in the nilotinib arms reached MMR and MR^4.5^ in a shorter time than those in the imatinib arm.

More patients in each nilotinib arm than in the imatinib arm achieved sustained DMR, both overall and independently of their Sokal risk group. The rates of TFR eligibility (estimated using ENESTfreedom criteria [18]) by 5 years with nilotinib were nearly double the rate achieved with imatinib, and although the rates increased in all 3 arms by 10 years, the imatinib arm remained below the nilotinib arms. The higher rate of sustained DMR by 10 years in patients with *BCR-ABL1*^IS^ ≤ 10% at 3 months versus those with *BCR-ABL1*^IS^ > 10% at 3 months, especially with nilotinib therapy, suggests that early molecular responses with nilotinib are associated with additional long-term benefits, including the possibility of attempting TFR.

Although mature PFS and OS rates were similar in all arms, including in patients who stayed on initial treatment, or were in follow-up or switched to second-line treatment during extension, subgroup analyses allowed identification of subsets of patients who had favorable outcomes. Rates of freedom from progression to AP/BP, OS, and PFS at 10 years among subsets of younger patients were numerically higher in both nilotinib arms versus the imatinib arm. In all 3 arms, the 10-year OS and PFS rates were substantially lower in the older patient subsets (showing poorer survival) than overall rates or rates in the younger patients (particularly in both nilotinib arms compared with imatinib), although these results should be interpreted with caution because the sample size was small for the older subset.

The overall frequency of nonhematologic AEs was similar with nilotinib and imatinib. Long-term follow-up confirms trends similar to those previously reported [5] in overall frequencies of AEs of interest for both nilotinib and imatinib.

Analyses of CVEs based on long-term follow-up showed a higher rate of CVEs with nilotinib versus imatinib. This result was consistent with earlier results from this study as well as other studies that have shown a risk of CVEs with second- and third-generation TKIs compared with imatinib [5, 20–22]. KM-estimated CVE rates were higher with nilotinib than with imatinib. Baseline Framingham general cardiovascular risk scores were predictive of a patient’s risk of developing CVEs with nilotinib therapy. In an analysis of newly occurring CVEs displayed by a yearly time window, new CVEs continued to occur in patients receiving nilotinib at similar rates each year (Supplementary Fig. 3). CVEs occurred more frequently among patients in the Framingham high-risk and intermediate-risk categories than in the low-risk category, and new events continued to occur throughout a long treatment period. In Framingham low-risk patients, the rates of CVEs after the first 5 years of treatment were higher on nilotinib than on imatinib, whereas patients in the low-risk category in all arms experienced few CVEs during the first 5 years [5]. Additionally, CVEs occurred more frequently in the nilotinib 400-mg twice-daily arm than in the other arms.

Despite specific efforts to address the key reversible risk factors, the risk of CVEs with nilotinib therapy beyond 5 years remains higher than with imatinib, including for patients in the Framingham low-risk category, who seem less tolerant to long-term treatment beyond 5 years. Hence, the benefit-risk balance for long-term treatment should be cautiously assessed, particularly in patients for whom TFR is not a feasible option. These patients must be informed on potential risks and closely monitored for any cardiovascular comorbidities during treatment with nilotinib.

Overall, efficacy and safety results from ENESTnd support the use of nilotinib 300-mg twice-daily as frontline therapy for patients with CML-CP for optimal long-term outcomes, including CML-related deaths, which continued to be numerically lower with nilotinib versus imatinib after 10 years of treatment. Further, no CML-related deaths were reported in the nilotinib 300-mg twice-daily arm beyond 5 years.

The positive benefit-risk balance is especially notable in the context of TFR as a treatment goal. The estimated rates of TFR eligibility were higher with nilotinib (at 5 and 10 years) than with imatinib, suggesting that frontline nilotinib therapy may be a superior choice for patients looking to achieve deeper molecular responses needed for attempting TFR or for patients who are aiming to achieve optimal long term outcomes in CML.

## Supplementary information

Supplement

## Data Availability

Novartis is committed to sharing with qualified external researchers, access to patient-level data and supporting clinical documents from eligible studies. These requests are reviewed and approved by an independent review panel on the basis of scientific merit. All data provided are anonymized to respect the privacy of patients who have participated in the trial in line with applicable laws and regulations. This trial data availability is according to the criteria and process described on www.clinicalstudydatarequest.com.
