# Supplementary material for: Long-term outcomes with frontline nilotinib versus imatinib in newly diagnosed chronic myeloid leukemia in chronic phase: ENESTnd 10-year analysis
Source: Leukemia. 2021 Jan 7;35(2):440–53. doi: 10.1038/s41375-020-01111-2 (PMC7862065; doi:10.1038/s41375-020-01111-2)
Supplement: Supplementary file 1 — Supplement [file 41375_2020_1111_MOESM1_ESM.docx]

**Supplementary Appendix**

**Supplemental Figure 1.** CONSORT diagram for ENESTnd 10-year analysis. LPLV date, 21 August 2019.


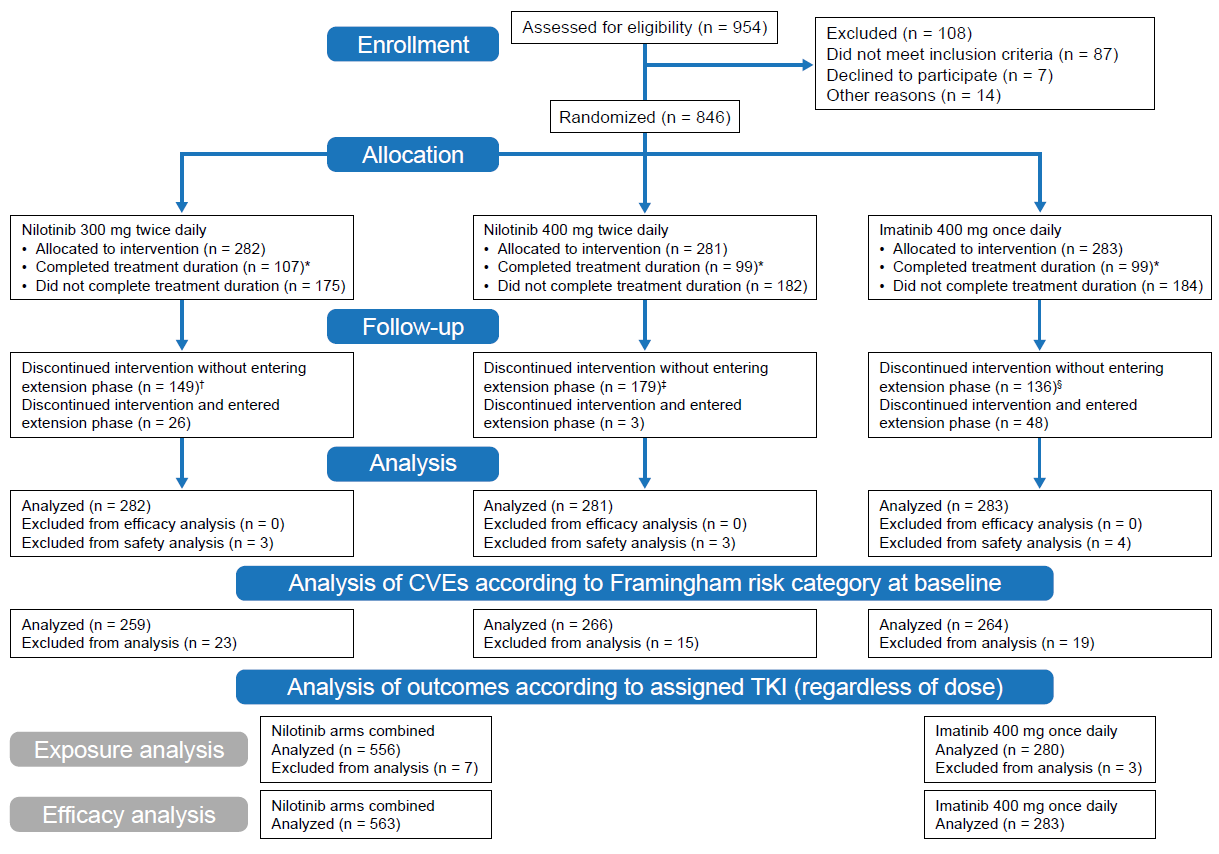


Efficacy analyses, including molecular response rates, were based on all randomized patients (intent-to-treat population). Safety analyses were based on patients who received ≥ 1 dose of study treatment.

LPLV indicates last patient’s last visit.

*Duration of study follow-up was ≥ 10 years.

†Reasons for discontinuation: AEs (n = 53), abnormal laboratory values (n = 9), withdrawal of consent (n = 29), death (n = 9), disease progression (n = 2), suboptimal response or treatment failure (n = 11), and other^||^ (n = 36). On discontinuation, 26 patients entered the extension phase. Two of these patients entered the extension phase to receive imatinib 400 mg once daily, and 24 patients received nilotinib 400 mg twice daily.

‡Reasons for discontinuation: AEs (n = 89), abnormal laboratory values (n = 9), withdrawal of consent (n = 34), death (n = 3), disease progression (n = 4), suboptimal response or treatment failure (n = 13), and other^||^ (n = 27). On discontinuation, 3 patients entered the extension phase. All 3 patients entered extension phase to receive imatinib 400 mg once daily.

§Reasons for discontinuation: AEs (n = 43), abnormal laboratory values (n = 3), withdrawal of consent (n = 31), death (n = 3), disease progression (n = 10), suboptimal response or treatment failure (n = 19), and other^||^ (n = 27). On discontinuation, 48 patients entered the extension phase. All 48 patients entered extension phase to receive nilotinib 400 mg twice daily.

||Other includes abnormal test procedure results, patient’s condition no longer required study drug, loss to follow-up, administrative problems, and protocol deviation.

**Supplemental Figure 2.** Kaplan-Meier estimated (A) progression-free survival and (B) overall survival.

**
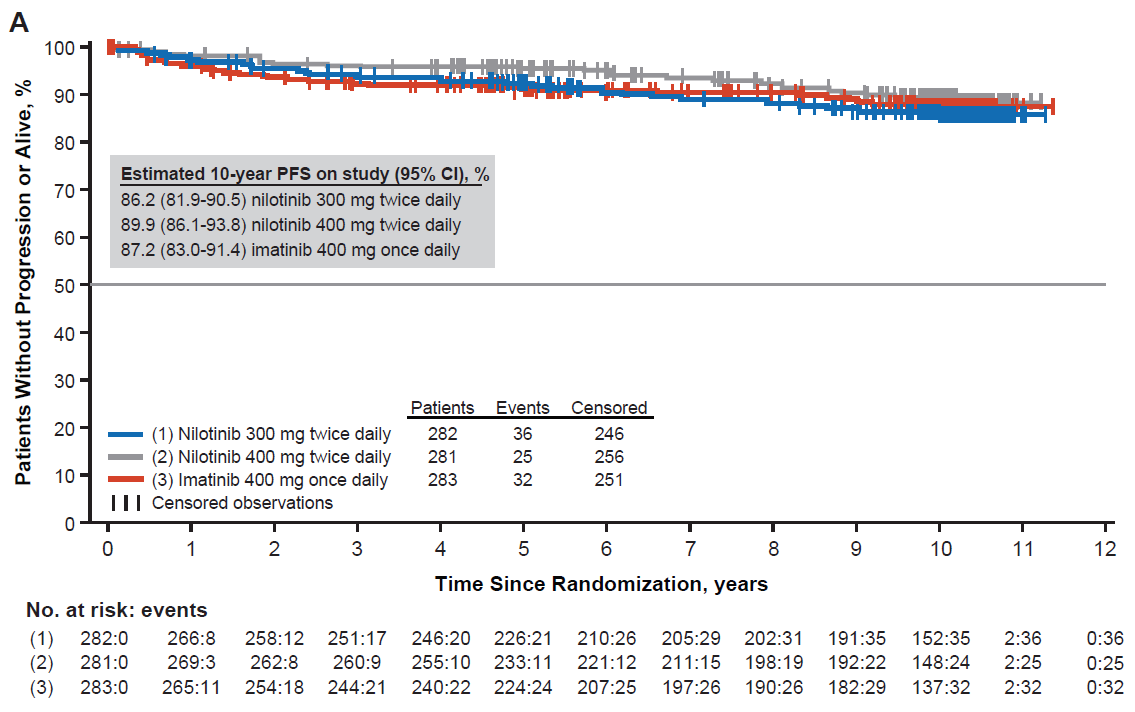
**

**
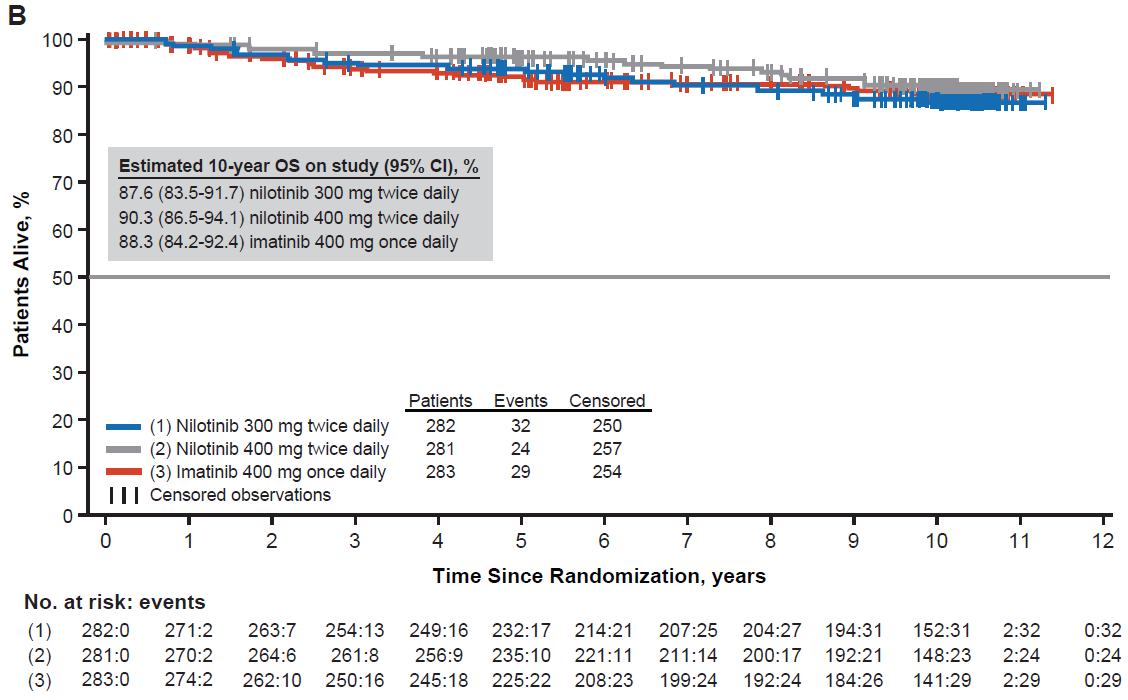
**

**Supplemental Figure 3.** Cardiovascular events (CVEs) by year. Rates of CVEs occurring in year 1 and newly occurring CVEs in the following years. Denominators are the number of patients on treatment during the indicated years. Note: The incidence of certain CVEs appears higher in later years compared with previous years owing to a lower number of patients on treatment.

**
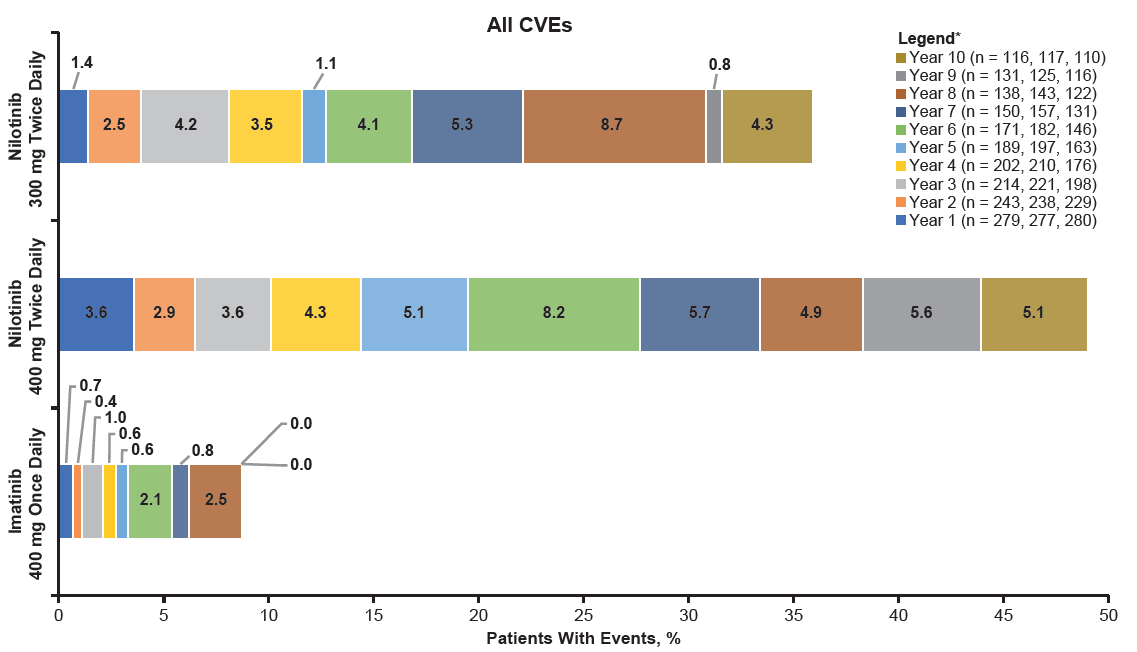
**

*Listed n values are the number of on-treatment patients at the beginning of the indicated year in the nilotinib 300-mg twice-daily, nilotinib 400-mg twice-daily, and imatinib arms, respectively, and are used as the denominator for calculating the percentage of patients with events in each year.

**Supplemental Table 1.** Patient disposition.

| Patient status, n (%) | Nilotinib 300 mg twice daily  n = 282 | Nilotinib 400 mg twice daily  n = 281 | Imatinib 400 mg once daily  n = 283 |
| --- | --- | --- | --- |
| Treatment duration completed per protocol* | 107 (37.9) | 99 (35.2) | 99 (35.0) |
| Discontinued core treatment without entering extension phase | 149 (52.8) | 179 (63.7) | 136 (48.1) |
| Discontinuation reason |  |  |  |
| Adverse event(s) | 53 (18.8) | 89 (31.7) | 43 (15.2) |
| Abnormal laboratory value(s) | 9 (3.2) | 9 (3.2) | 3 (1.1) |
| Withdrawal of consent | 29 (10.3) | 34 (12.1) | 31 (11.0) |
| Death | 9 (3.2) | 3 (1.1) | 3 (1.1) |
| Disease progression | 2 (0.7) | 4 (1.4) | 10 (3.5) |
| Suboptimal response or treatment failure^†^ | 11 (3.9) | 13 (4.6) | 19 (6.7) |
| Treatment failure^‡^ | 3 (1.1) | 4 (1.4) | 3 (1.1) |
| Suboptimal response^‡^ | 5 (1.8) | 4 (1.4) | 15 (5.3) |
| Did not meet study criteria for suboptimal response or treatment failure^§^ | 3 (1.1) | 5 (1.8) | 1 (0.4) |
| Other^\|\|^ | 36 (12.8) | 27 (9.6) | 27 (9.5) |
| Discontinued core treatment and entered extension phase | 26 (9.2) | 3 (1.1) | 48 (17.0) |
| Discontinuation reason |  |  |  |
| Disease progression | 0 | 0 | 2 (0.7) |
| Suboptimal response or treatment failure^†^ | 26 (9.2) | 3 (1.1) | 46 (16.3) |
| Treatment failure^‡^ | 5 (1.8) | 1 (0.4) | 20 (7.1) |
| Suboptimal response^‡^ | 18 (6.4) | 2 (0.7) | 22 (7.8) |
| Did not meet study criteria for suboptimal response of treatment failure^§^ | 3 (1.1) | 0 | 4 (1.4) |

ELN indicates European LeukemiaNet.

*Duration of study follow-up was ≥ 10 years.

†Patients with suboptimal response or treatment failure during core phase could discontinue core phase and enter either extension or follow-up phase. Imatinib patients could dose escalate first for suboptimal response or treatment failure and could then enter the extension phase and receive nilotinib 400-mg twice daily. Nilotinib patients could enter the extension phase and receive either imatinib 400 mg twice daily (only until 36-month protocol amendment) or nilotinib 400 mg twice daily (applicable only for patients randomized to nilotinib 300 mg twice daily group) for suboptimal response (300-mg twice-daily arm only) or treatment failure (300-mg twice-daily arm and 400-mg twice-daily arm).

‡Met ELN criteria based on actual assessments.

§Did not meet modified ELN criteria for suboptimal response or treatment failure; discontinued due to suboptimal response or treatment failure per investigator assessment.

||Other reasons included abnormal test procedure result(s), patient’s condition no longer required study drug, loss to follow-up, administrative problems, and protocol deviation.

**Supplemental Table 2.** Most frequent causes of death by time interval (≥ 2 deaths in any treatment arm).

| n | Nilotinib 300 mg twice daily n = 282 | Nilotinib 400 mg twice daily n = 281 | Imatinib 400 mg once daily n = 283 |
| --- | --- | --- | --- |
| Overall on study* | 32 | 23^†^ | 29 |
| < 5 years | 16 | 9 | 18 |
| CML | 6 | 4 | 12 |
| Cardiac disorders^‡^ | 2 | 0 | 0 |
| General disorders | 3 | 1 | 0 |
| Infections and infestations | 1 | 1 | 5 |
| Neoplasm benign, malignant and unspecified^‡^ | 1 | 2 | 0 |
| Other^\|\|^ | 3 | 1 | 1 |
| > 5 years | 16 | 14 | 11 |
| CML | 0 | 1 | 3 |
| Cardiac disorders^‡^ | 2 | 2 | 1 |
| General disorders | 3 | 5 | 2 |
| Infections and infestations | 4 | 2 | 3 |
| Neoplasm benign, malignant and unspecified^¶^ | 4 | 3 | 1 |
| Other^#^ | 3 | 1 | 1 |

*Overall, 84 deaths occurred on study.

†One death occurred after randomization and prior to first dose and hence is not reported in the table.

‡Includes acute myocardial infarction, myocardial infarction, cardiac arrest, and cardiogenic shock.

‡Includes ovarian epithelial cancer, gastric cancer, and metastatic neoplasm.

||Includes gastrointestinal disorders, injury, poisoning and procedural complications, nervous system disorders, psychiatric disorders, and renal and urinary disorders.

¶Includes gastric adenocarcinoma, metastatic malignant melanoma, myelodysplastic syndromes, neuroblastoma, pancreatic carcinoma, plasma cell myeloma, and rectal cancer.

#Includes vascular disorders, immune system disorders, nervous system disorders, and respiratory disorder.

**Supplemental Table 3.** Achievement of sustained DMR by 10 years.

| Patients, n/N (%)* | Nilotinib 300 mg twice daily | Nilotinib 400 mg twice daily | Imatinib 400 mg once daily |
| --- | --- | --- | --- |
| Total | 138/282 (48.9) | 135/281 (48.0) | 89/283 (31.4) |
| By Sokal risk at diagnosis |  |  |  |
| Low | 52/103 (50.5) | 58/103 (56.3) | 41/104 (39.4) |
| Intermediate | 55/101 (54.5) | 49/100 (49.0) | 30/101 (29.7) |
| High | 31/78 (39.7) | 28/78 (35.9) | 18/78 (23.1) |
| By early molecular response |  |  |  |
| *BCR-ABL1*^IS^ ≤ 10% at 3 months | 135/234 (57.7) | 127/232 (54.7) | 77/176 (43.8) |
| *BCR-ABL1*^IS^ > 10% at 3 months | 2/24 (8.3) | 7/28 (25.0) | 11/88 (12.5) |
| By time to first MR^4.5^ |  |  |  |
| ≤ year 2 | 55/70 (78.6) | 43/53 (81.1) | 23/25 (92.0) |
| > year 2 to ≤ year 5 | 70/81 (86.4) | 75/94 (79.8) | 53/64 (82.8) |
| > year 5 to ≤ year 9 | 13/20 (65.0) | 17/24 (70.8) | 11/20 (55.0) |

Sustained DMR was defined as MR^4.5^ achieved during ≥ 2 years of TKI treatment followed by 1 year with no assessment worse than MR^4^, ≤ 2 assessments between MR^4^ and MR^4.5^, and MR^4.5^ in the last assessment (with assessments every 3 or 6 months) per the criteria for TFR eligibility in the ENESTfreedom study.^18^

*In each subgroup, N (denominator) is the number of patients in the subgroup, n is the number of patients who achieved sustained DMR by year 10 among N, and % is calculated from n/N.

**Supplemental Table 4.** Adverse events (regardless of relationship to study drug) and newly occurring or worsening hematologic and biochemical laboratory abnormalities.

|  | **Any grade** | | | **Grade 3/4** | | |
| --- | --- | --- | --- | --- | --- | --- |
|  | Nilotinib 300 mg twice daily  n = 279 | Nilotinib 400 mg twice daily  n = 277 | Imatinib 400 mg once daily  n = 280 | Nilotinib 300 mg twice daily  n = 279 | Nilotinib 400 mg twice daily  n = 277 | Imatinib 400 mg once daily  n = 280 |
| Any AE, n (%) | 278 (99.6) | 274 (98.9) | 278 (99.3) | 194 (69.5) | 218 (78.7) | 175 (62.5) |
| **AEs reported in ≥ 20% of patients in any arm, n (%)** | | | | | | |
| **Blood and lymphatic system disorders** | | | | | | |
| Thrombocytopenia | 54 (19.4) | 59 (21.3) | 53 (18.9) | 28 (10.0) | 33 (11.9) | 25 (8.9) |
| Anemia | 39 (14.0) | 48 (17.3) | 67 (23.9) | 16 (5.7) | 17 (6.1) | 20 (7.1) |
| Neutropenia | 45 (16.1) | 30 (10.8) | 59 (21.1) | 33 (11.8) | 25 (9.0) | 42 (15.0) |
| **Gastrointestinal disorders** | | | | | | |
| Nausea | 62 (22.2) | 88 (31.8) | 118 (42.1) | 7 (2.5) | 4 (1.4) | 4 (1.4) |
| Diarrhea | 59 (21.1) | 67 (24.2) | 135 (48.2) | 3 (1.1) | 7 (2.5) | 10 (3.6) |
| Abdominal pain upper | 52 (18.6) | 64 (23.1) | 44 (15.7) | 3 (1.1) | 1 (0.4) | 2 (0.7) |
| Vomiting | 47 (16.8) | 60 (21.7) | 79 (28.2) | 2 (0.7) | 4 (1.4) | 1 (0.4) |
| Constipation | 63 (22.6) | 53 (19.1) | 26 (9.3) | 2 (0.7) | 2 (0.7) | 0 |
| **General disorders and administration site conditions** | | | | | | |
| Fatigue | 69 (24.7) | 58 (20.9) | 57 (20.4) | 3 (1.1) | 4 (1.4) | 4 (1.4) |
| Peripheral edema | 34 (12.2) | 43 (15.5) | 63 (22.5) | 2 (0.7) | 0 | 0 |
| **Infections and infestations** | | | | | | |
| Nasopharyngitis | 81 (29.0) | 67 (24.2) | 65 (23.2) | 0 | 0 | 0 |
| Upper respiratory tract infection | 56 (20.1) | 67 (24.2) | 44 (15.7) | 1 (0.4) | 0 | 0 |
| **Musculoskeletal and connective tissue disorders** | | | | | | |
| Back pain | 64 (22.9) | 68 (24.5) | 55 (19.6) | 5 (1.8) | 7 (2.5) | 4 (1.4) |
| Arthralgia | 72 (25.8) | 66 (23.8) | 61 (21.8) | 1 (0.4) | 3 (1.1) | 1 (0.4) |
| Myalgia | 59 (21.1) | 56 (20.2) | 56 (20.0) | 2 (0.7) | 3 (1.1) | 1 (0.4) |
| Muscle spasms | 38 (13.6) | 37 (13.4) | 98 (35.0) | 0 | 2 (0.7) | 3 (1.1) |
| **Nervous system disorders** | | | | | | |
| Headache | 94 (33.7) | 105 (37.9) | 69 (24.6) | 9 (3.2) | 7 (2.5) | 2 (0.7) |
| **Respiratory, thoracic, and mediastinal disorders** | | | | | | |
| Cough | 55 (19.7) | 61 (22.0) | 41 (14.6) | 0 | 0 | 0 |
| **Skin and subcutaneous tissue disorders** | | | | | | |
| Rash | 110 (39.4) | 125 (45.1) | 58 (20.7) | 3 (1.1) | 7 (2.5) | 5 (1.8) |
| Alopecia | 42 (15.1) | 58 (20.9) | 21 (7.5) | 0 | 0 | 0 |
| Pruritus | 61 (21.9) | 56 (20.2) | 20 (7.1) | 2 (0.7) | 1 (0.4) | 0 |
| **Vascular disorders** | | | | | | |
| Hypertension | 45 (16.1) | 56 (20.2) | 17 (6.1) | 5 (1.8) | 13 (4.7) | 2 (0.7) |
| **Grade 3/4 hematologic abnormalities reported in ≥ 5% of patients in any arm, n (%)** | | | | | | |
| Decreased absolute lymphocytes | NA | NA | NA | 36 (12.9) | 22 (7.9) | 42 (15.0) |
| Decreased absolute neutrophils | NA | NA | NA | 34 (12.2) | 32 (11.6) | 62 (22.1) |
| Decreased hemoglobin | NA | NA | NA | 12 (4.3) | 14 (5.1) | 23 (8.2) |
| Decreased total WBC | NA | NA | NA | 9 (3.2) | 10 (3.6) | 29 (10.4) |
| Decreased platelet count (direct) | NA | NA | NA | 29 (10.4) | 35 (12.6) | 27 (9.6) |
| **Grade 3/4 biochemical abnormalities reported in ≥ 5% of patients in any arm, n (%)** | | | | | | |
| Increased total bilirubin | NA | NA | NA | 12 (4.3) | 25 (9.0) | 1 (0.4) |
| Increased glucose | NA | NA | NA | 24 (8.6) | 21 (7.6) | 1 (0.4) |
| Increased lipase (blood) | NA | NA | NA | 29 (10.4) | 30 (10.8) | 12 (4.3) |
| Decreased phosphate (inorganic phosphorus) | NA | NA | NA | 24 (8.6) | 31 (11.2) | 37 (13.2) |
| Increased ALT | NA | NA | NA | 12 (4.3) | 27 (9.7) | 7 (2.5) |

ALT indicates alanine aminotransferase; NA, not applicable; WBC, white blood cell.

**Supplemental Table 5. Adverse events of interest (regardless of relationship to study drug)**

|  | **Any grade** | | | **Grade 3/4** | | |
| --- | --- | --- | --- | --- | --- | --- |
| **n (%)** | Nilotinib 300 mg twice daily  n = 279 | Nilotinib 400 mg twice daily  n = 277 | Imatinib 400 mg once daily  n = 280 | Nilotinib 300 mg twice daily  n = 279 | Nilotinib 400 mg twice daily  n = 277 | Imatinib 400 mg once daily  n = 280 |
| Cardiac failure | 9 (3.2) | 7 (2.5) | 4 (1.4) | 3 (1.1) | 3 (1.1) | 3 (1.1) |
| Electrocardiogram QT prolongation | 19 (6.8) | 22 (7.9) | 11 (3.9) | 10 (3.6) | 5 (1.8) | 5 (1.8) |
| Blood cholesterol increased | 47 (16.8) | 48 (17.3) | 4 (1.4) | 1 (0.4) | 3 (1.1) | 0 |
| Blood glucose increased | 35 (12.5) | 36 (13.0) | 8 (2.9) | 7 (2.5) | 10 (3.6) | 0 |
| Fluid retention | 66 (23.7) | 75 (27.1) | 167 (59.6) | 11 (3.9) | 9 (3.2) | 7 (2.5) |
| Severe fluid retention | 6 (2.2) | 5 (1.8) | 7 (2.5) | 2 (0.7) | 1 (0.4) | 0 |
| Edema and other fluid retentions | 63 (22.6) | 73 (26.4) | 167 (59.6) | 9 (3.2) | 8 (2.9) | 7 (2.5) |
| Rash | 120 (43.0) | 139 (50.2) | 69 (24.6) | 3 (1.1) | 7 (2.5) | 7 (2.5) |
| Significant bleeding | 11 (3.9) | 20 (7.2) | 11 (3.9) | 3 (1.1) | 7 (2.5) | 4 (1.4) |
| CNS hemorrhage | 2 (0.7) | 3 (1.1) | 2 (0.7) | 1 (0.4) | 1 (0.4) | 2 (0.7) |
| Gastrointestinal hemorrhage | 9 (3.2) | 18 (6.5) | 9 (3.2) | 2 (0.7) | 6 (2.2) | 2 (0.7) |
| Thrombocytopenia | 60 (21.5) | 66 (23.8) | 59 (21.1) | 32 (11.5) | 36 (13.0) | 28 (10.0) |
| Hepatotoxicity | 135 (48.4) | 147 (53.1) | 49 (17.5) | 24 (8.6) | 46 (16.6) | 14 (5.0) |
| Elevation of transaminase and bilirubin | 132 (47.3) | 143 (51.6) | 45 (16.1) | 24 (8.6) | 41 (14.8) | 13 (4.6) |
| Drug induced liver injury | 6 (2.2) | 16 (5.8) | 8 (2.9) | 1 (0.4) | 5 (1.8) | 2 (0.7) |
| Pancreatitis | 5 (1.8) | 10 (3.6) | 2 (0.7) | 1 (0.4) | 5 (1.8) | 0 |
| Renal event | 8 (2.9) | 11 (4.0) | 11 (3.9) | 1 (0.4) | 5 (1.8) | 5 (1.8) |

CNS indicates central nervous system; MedDRA, Medical Dictionary for Regulatory Activities.

All events refer to predefined groupings of MedDRA preferred terms or standardized MedDRA queries. Patients with multiple events for a given AE term or category were counted only once for the AE term or category. CVEs are reported in Table 4.
